# The Impact of Dose in an mHealth Intervention to Support Parents and Carers Via Healthy Beginnings for Hunter New England Kids Program: Pragmatic Randomized Controlled Trial

**DOI:** 10.2196/70158

**Published:** 2025-10-01

**Authors:** Alison L Brown, Nayerra Hudson, Jessica Pinfold, Rebecca Sewter, Lynda Davies, Christophe Lecathelinais, Jacklyn K Jackson, Tessa Delaney, Sienna Kavalec, Rachel Sutherland

**Affiliations:** 1 Population Health, Hunter New England Local Health District, Booth Building, Longworth Avenue, Newcastle, New South Wales, 2287, Australia, 61 0403702433; 2 School of Medicine and Public Health, University of Newcastle, University Drive, Callaghan, Australia, 61 49246000; 3 Hunter Medical Research Institute, Kookaburra Circuit, Newcastle, Australia, 61 4042 0000; 4 National Centre of Implementation Science, Newcastle, Australia; 5 Centre for Population Health, New South Wales Ministry of Health, St Leonards, Australia

**Keywords:** child health, infants, mHealth, mobile health, digital health, text messaging, dose, engagement

## Abstract

**Background:**

The dose of mobile health (mHealth) interventions can influence participant engagement, acceptability, and overall impact. However, few mHealth interventions have explored this dose-response relationship.

**Objective:**

This study aims to explore how dose influences the acceptability, engagement, cost, and impact on infant feeding status of a parent-targeted mHealth text messaging program which aims to enhance child health, including breastfeeding exclusivity and duration.

**Methods:**

This pragmatic randomized controlled trial was conducted from October 2021 to May 2024. The Healthy Beginnings for Hunter New England Kids (HB4HNEKids) program provides- text messages aimed to support parents and carers and their children by providing evidence-based preventive health information across the first 2000 days. Participants were enrolled in HB4HNEKids from 5 Child and Family Health Services in the Hunter New England region of New South Wales, Australia, and randomized into either a high-dose or low-dose text message group for the first 2 years of the pilot program. Dose refers to the quantity and frequency of text messages sent to participants. Participants in the high-dose text message group received an average of 111‐121 text messages, and the low-dose text message group received 80‐82 text messages across the 2 years. Outcomes of interest included acceptability, engagement, cost, and infant feeding status in relation to dose. Engagement with the messages was determined using click rates and program opt-out rates. Participant acceptability was assessed via a brief survey. Impact on infant feeding status (ie, breastfeeding, formula feeding, or mixed feeding) was determined by participants reporting their feeding status at several time points across the program. Cost was determined by assessing the per participant and total cost of sending text messages for each dose group across the 2-year period.

**Results:**

There were no statistically significant differences in click rates between high or low-dose text message groups. In the first 6 months, significantly more participants opted out of the high-dose text message group (191/2724; 7%) compared to the low-dose (108/2812; 3.8%*; P*<.001). In terms of program acceptability, 183 out of 214 (85.5%) participants of the high-dose and 228 out of 252 (90.5%) participants of the low-dose text message group were satisfied with the frequency of text messages. In addition, 188 out of 215 (87%) participants of high-dose and 220 out of 255 (86%) participants of low-dose text message group indicated they would recommend the program to other caregivers. The average per participant and total cost to the health service for sending messages was lower in the low-dose group (A$9.32 per participant and A$15,271.48 total; A$1 is approximately equal to US $0.68) compared to the high-dose text message group (A$12.96 per participant and A$21,241.44 total).

**Conclusions:**

The HB4HNEKids program demonstrated positive outcomes including high acceptability across both groups and no impact on infant feeding status, irrespective of dose. Given the higher opt-out rates and message costs in the high-dose text message group, a lower dose is likely more scalable for future use.

## Introduction

The first 2000 days of life (conception to 5 years) is a key period for child physical, cognitive, social, and emotional development and can have significant lifelong impacts [1]. Therefore, the first 2000 days is an important period to enhance a child’s overall health. Accordingly, New South Wales (NSW) Health has developed a strategic policy to support families in the first 2000 days, stipulating the provision of care to all [1]. Currently, NSW Child and Family Health Services (CFHS) offer families routine health and development checks over their child’s first 2000 days, via face-to-face home or clinic visits which target key age and stage related milestones. However, engagement with the health service by families is fragmented. Key factors that impact the provision of care by health services include limited staff capacity, while parent uptake of services is influenced by the need for convenient and accessible services that align with family routines and roles [2].

Mobile health (mHealth) is considered a promising service delivery model to support families during the first 2000 days [3,4]. A systematic review of mHealth interventions delivered via text messages or telephone to prevent early childhood obesity found that over 60% of the studies reported improvements in 1 or more key health-related behaviors, including breastfeeding, introduction of solids, physical activity, screen time, sleep, and tummy time [5]. This highlights the delivery of mHealth interventions as a promising population-level approach. mHealth text message-based interventions have the ability to provide evidence-based information and additional care pathways at scale, while being cost-effective [5-7]. mHealth infrastructure is currently being used by CFHS in the scheduling of appointments; however, it is not routinely used to support service delivery activities, including providing evidence-based information to parents and carers. As such, an mHealth program, Healthy Beginnings for Hunter New England Kids (HB4HNEKids), was developed that aims to support parents and carers with the provision of evidence-based age and stage-appropriate care, in the form of text messages, across the first 2000 days. The program was piloted across a 3-year period (October 2021-July 2024) to test the feasibility of program methods and test any adjustments required to improve the quality of the program. The program is now offered as part of usual care for CFHS and is currently being rolled out throughout the Hunter New England (HNE) region of NSW, Australia. However, as part of the pilot and ongoing rollout of HB4HNEKids, routine quality assurance data has been collected to monitor and improve program reach, engagement, and overall health outcomes.

Dose is an important aspect of any mHealth intervention, particularly long-term programs such as HB4HNEKids. The dose of a program can have a direct effect on participant engagement, and therefore the effectiveness of an intervention [8]. However, there are limited mHealth studies that have examined the dose-response relationship as part of program evaluation to assess its effect on acceptability, engagement, impact, and cost [9]. Furthermore, there is a scarcity of studies that have looked at the dose-response relationship in long-term studies [9]. Initial investigation into the acceptability of text message frequency for an intervention targeting preschool parents identified significant variability in what was an acceptable frequency of messages across the school year, with the study reporting 1 text message per week was too little and 5 text messages were excessive [10]. This highlights that dose can have a direct response on engagement with mHealth programs and must be a key consideration in implementation planning.

With minimal evidence regarding optimal dose in parent-targeted mHealth (text message) interventions, this study aims to explore how dose influences the acceptability, engagement, impact on infant feeding status, and cost of a parent-targeted mHealth text messaging program, HB4HNEKids.

## Methods

### Setting

This trial was a pragmatic randomized controlled trial. The program was offered as part of routine service delivery provided by CFHS in the HNE region of NSW, as part of a pilot feasibility trial. The HNE region covers 131,785 km^2^ and has an estimated population of 973,653 people [11,12]. In 2020, there were 10,242 births within the HNE Local Health District, accounting for approximately 10% of births in NSW [13]. There are approximately 150 CFHS staff supporting parents and carers (hereafter referred to as parents) across HNE with the primary responsibility of comprehensively assessing and facilitating care pathways that best support the physical, developmental, psychological, and socio-economic health and well-being of families with children aged 0‐5 years [14].

### Participants and Recruitment

#### Child and Family Health Services

A sample of 5 CFHS in the HNE region was selected for inclusion in the pilot feasibility trial of HB4HNEKids. Recruitment of CFHS was identified through an expression of interest process, with services volunteering for study participation. To ensure representation across a variety of population groups and geographical areas, the research team sought to include CFHS that met at least 1 of each of the following eligibility criteria:

1. Aboriginal CFHS (n=2)
2. Rural or remote service CFHS (n=1; based on a population size of less than 50,000)
3. Regional CFHS (n=1; based on a population size between 50,000‐100,000)
4. Metropolitan CFHS (n=1; based on a population size of more than 100,000)

#### Parents and Carers

As per existing health service procedures, CFHS receive a referral from public and private maternity services following the birth of a baby [14]. Upon discharge, staff from one of the 5 HNE CFHS contacted the parent (participants) via phone call or text message to offer a 1‐4 week health and development check. Parents within the catchment area of the pilot CFHS sites were offered HB4HNEKids as part of routine care. During the initial contact to schedule families into a 1‐4 week health and development check, CFHS staff confirmed feeding status (ie, if the child was breastfeeding, mixed feeding, formula feeding), child name, mother mobile number, and “offer of service” (ie, yes, client refused, or unable to contact after multiple attempts). If a staff member was unable to determine infant feeding status during the initial contact, they would locate the “infant feeding status” via the hospital discharge summary provided by maternity services. No other participant characteristics were collected during this study.

### Healthy Beginnings for HNEKids Program

HB4HNEKids is a text message program (model of care) that targets barriers and enablers to recommended infant feeding practices, child nutrition, physical activity, small screen recreation, child development, parent well-being and primary health checks, and immunizations. One of the program’s primary outcomes is to increase the duration of any breastfeeding, in alignment with international recommendations. The mHealth program was developed collaboratively with a multidisciplinary team including CFHS, multicultural health, Aboriginal partners, allied health professionals, and end-users (parents). The program has been adapted from the Healthy Beginnings CHAT study [15]. The optimized text messages used the behavior change wheel [16] and Theoretical Domains Framework [17] to develop message content and support link selection. The text messages aimed to provide brief content (mostly <160 characters) with clickable links to evidence-based “online” videos, fact sheets, websites, and relevant support services. Text message content is aligned to the age, stage, and developmental milestones of the child across the first 2000 days. Participants receive different text messages based on their reported infant feeding status (breastfeeding, mixed feeding, or formula feeding) that are tailored to support their child’s needs. Example text messages can be found in Table 1. While the HB4HNEKids program aims to provide support across the first 2000 days, this study focuses specifically on the first 2 years. Outcome data on the effectiveness of HB4HNEKids will be reported elsewhere.

**Table 1.** Example text messages for Healthy Beginnings for Hunter New England Kids.

Days	Phases	Messages
Day 9	1 (day 3 to 6 months)	Ensuring bub is attached well helps reduce discomfort. Good attachment signs are mouth open wide, chin to breast, good suck & swallow.
Day 11	1 (day 3 to 6 months)	Breastfeeding takes practice. Give yourself time to try different positions & find what works best for you & #firstname#.
Day 15	1 (day 3 to 6 months)	There are simple ways to know if #firstname# is getting enough milk such as 5‐6 wet nappies/day.
Day 47	1 (day 3 to 6 months)	Intending to express? Hand expressing breastmilk can be done anywhere. Practice at home so you're comfortable.
Day 192	2 (6‐12 months)	There are 4 easy steps to healthy teeth & gums. Start brushing twice a day when #firstname# first teeth appear.
Day 334	2 (6-12 months)	Continuing to provide breastmilk beyond 12 months is great for you & #firstname#. If you’re both enjoying it you don’t have to stop, but if you are looking to introduce full cream milk you can from 12 months. For any questions contact your CFHN or GP.
Day 403	3 (1‐2 years)	Managing your emotions is important so you can enjoy parenting & a happy home for you & your family.
Day 475	3 (1‐2 years)	#firstname#does not need any screen time. Video chats with family & friends are ok. Try reading, singing, puzzles & craft instead.
Day 524	3 (1‐2 years)	Remember, offering new foods many times (up to 15) gives #firstname# a chance to learn about their smell, touch & taste.

The program was broken up into four phases across the first 2 years of the program as follows: phase 0 (day 0-2; onboarding); phase 1 (day 3 to 6 months); phase 2 (6‐12 months); and phase 3 (1‐2 years).

### Delivery of Messages

Participants were sent text messages to their nominated mobile number between the hours of 9 AM to 12 PM across the week according to the message schedule using the SMS platform, DirectSMS. Each text message unit (n<160 characters) had an associated cost to the health service of 13.5 cents (A$0.135; A$1 is approximately equal to US $0.68) per unit. Participants were provided a link to opt-out of the program if they wanted to on day 1, 51, 296, and 461.

### Dose Randomization

#### Overview

Participants were randomized into one of 2 intervention groups (high or low dose text message group) in randomly sequenced blocks of 2 and 4 using a random number function set up by an independent statistician in SAS 9.3. As the intervention content varied by feeding status, the randomization was stratified by the feeding status (breastfed vs mixed fed vs formula fed) provided at the initial contact with CFHS. CFHS staff and participants were blinded to group allocation.

#### Low-Dose Text Message Group

The frequency and dose of text messages in the low-dose text message group were adapted from the “Healthy Beginnings” CHAT study [15]. The low-dose text message group received up to 2 messages per week in the first month and approximately 1 per week from 2 months to 2 years ( Table 2).

**Table 2.** Number of messages sent by study phase, feeding status, and dose group.

Phases		Number of messages by group	
		High-dose	Low-dose
Phase 0 (0‐2 days)			
Breastfeeding and mixed feeding		1	1
Formula feeding		1	1
Phase 1 (2 days to 6 months)			
Breastfeeding		40	24
Mixed feeding		38	23
Formula feeding		31	23
Phase 2^a^ (6‐12 months)			
Breastfeeding and mixed feeding		26	26
Formula feeding		25	25
Phase 3 (1‐2 years)			
Breastfeeding and mixed feeding		54	31
Formula feeding		54	31
Total			
Breastfeeding		121	82
Mixed feeding		119	81
Formula feeding		111	80

a dose was not applied to phase 2 (6‐12 months)

#### High-Dose Text Message Group

The high-dose text message group received up to 3‐4 messages per week in the first month, 2 per week in the second month and 5 per month from 3 months to 2 years. The number of messages was determined by the number of identified barriers that were considered to impact behavioral outcomes.

### Data Collection

#### Infant Feeding Status

As part of routine service delivery, participants were asked to respond to a message asking how they were feeding their child or children at days 21 (3 weeks), 97 (3 months), 169 (6 months), 275 (9 months), 360 (1 year), and 552 (18 months) to determine any change to feeding status. Participants who entered the study as formula feeding received the day 21 message prompt, and anyone flagged as formula feeding stopped receiving follow-up prompts. Possible feeding status responses included: breastmilk, mixed feeding, and formula. If the feeding status changed, the message content was tailored according to their new feeding status. To calculate feeding rates and breastfeeding outcomes across the lifespan of the program, “assumed” feeding status was used whereby the participant’s feeding status was carried over to the next phase of the program unless participants provided an update to their feeding status.

#### Engagement

Engagement with the messages was determined using the click rates from weblinks provided in each message, in addition to opt-out rates from the program.

##### Click Rates

Weblinks provided in the text messages were given a unique weblink shortener. Each weblink shortener was coded with the associated feeding status and dose, to enable the reporting of click rates by feeding status and dose. Click rates were calculated based on the number of unique times a link had been clicked divided by the number of participants that had been sent the corresponding link.

##### Opt-Out Rates

Participants were sent a message at day 1, 51, 296, and 461 that contained an opt-out link that participants had to click and provide their mobile number in order to opt-out. The opt-out link remained live for the course of the program, so that participants could click on the link at any point in time if they chose to no longer receive the program. Participants could also opt out via contact details provided, or by notifying CFHS. Opt-out rates were calculated by feeding status and dose.

##### Acceptability

At day 169 (approx. 5 months), participants were provided a link to a brief online survey to determine acceptability of the program. Participants were asked the following on a 5-point Likert scale (strongly agree to strongly disagree): (1) I found the Healthy Beginnings for HNEKids program acceptable; (2) Since I started the program, I have been happy with how often I have received the text messages; and (3) I would recommend the HB4HNEKids program to other caregivers.

### Cost

The average cost to the health service of sending 2 years of text messages per participant was collected for each group. This was calculated using the average number of text messages sent across the 3 infant feeding groups multiplied by the cost of sending a message via the SMS provider, DirectSMS. In addition, the total cost of sending the text messages was reported from the commencement of the program from October 2021 to May 2024, for each group.

### Statistics

Data was analyzed using the statistical software package SAS (version 9.4; SAS Institute). Descriptive statistics were used to describe outcomes such as the sample, cost, and acceptability measures. An intention to treat approach was used in the analyses of all study outcomes, whereby analysis occurred according to the randomized treatment allocation (dose group). Between-group differences in percentages were examined using Chi-square tests for each categorical outcome. The comparisons of interest included feeding status versus dose after each message feeding prompt, click rates versus dose stratified by feeding status, and opt-out rates versus dose during each study phase. Analyses of the outcomes used a 2-tailed test with an alpha level of 5%.

### Ethical Considerations

Ethical approval to undertake the study was obtained from the Hunter New England Human Research Ethics Committee (23/12/13/4.10) and the University of Newcastle Human Research Ethics Committee (H-2024‐0025). This research was conducted in compliance with informed consent guidelines and adhered to national law and regulations regarding the protection of personal information, privacy, and human rights. This trial has been retrospectively registered in Australian New Zealand Clinical Trial Registry (ACTRN12625000574448; registered June 3, 2025).

## Results

### Text Messages

**Figure 1.**
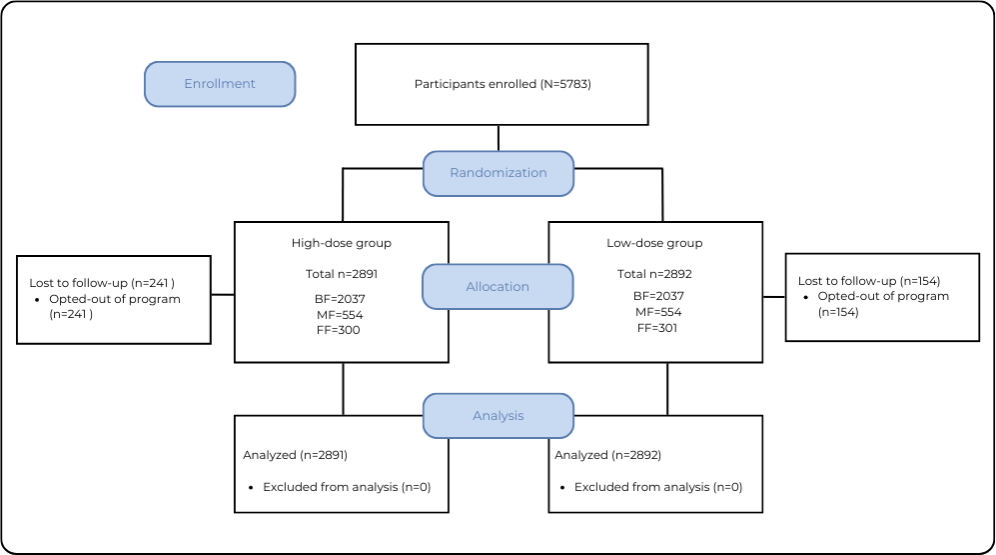
CONSORT (Consolidated Standards of Reporting Trials) flow diagram. BF: breastfeeding; MF: mixed feeding; FF: formula feeding.

From October 2021 to May 2024, 5783 participants were engaged in the program (Figure 1). In total, 307,726 messages were sent to participants with children between 0‐2 years old. Table 2 shows the number of messages sent to each parent, in each phase of the program by dose and feeding status.

### Feeding Status Responses

Of those that responded at least once with their feeding status, there were no statistically significant differences in feeding status between high and low dose text message groups, as seen in Table 3.

**Table 3.** Feeding status by dose.

Age of child (months) and feeding status		High-dose group, n (%)	Low-dose group, n (%)	Difference between groups (*P* value)
1 month				.74
Breastfeeding		1763 (64.8)	1760 (64.1)	
Mixed feeding		580 (21.3)	583 (21.3)	
Formula feeding		378 (13.9)	401 (14.6)	
3 months				.64
Breastfeeding		1280 (58.9)	1346 (59.4)	
Mixed feeding		414 (19.1)	447 (19.7)	
Formula feeding		478 (22)	474 (20.9)	
5 months				.29
Breastfeeding		1028 (54.5)	1061 (54.1)	
Mixed feeding		344 (18.2)	394 (20.1)	
Formula feeding		514 (27.3)	507 (25.8)	
9 months				.11
Breastfeeding		610 (46.6)	629 (46.2)	
Mixed feeding		219 (16.7)	267 (19.6)	
Formula feeding		481 (36.7)	465 (34.2)	
12 months				.10
Breastfeeding		502 (43.4)	510 (42.4)	
Mixed feeding		175 (15.1)	221 (18.4)	
Formula feeding		479 (41.4)	471 (39.2)	

### Engagement

#### Click Rates

Table 4 outlines the click rates for the HB4HNEKids program. Overall, there was a total click rate on weblinks of 16.3% (47,369/290,771) across both doses. There were no statistically significant differences in click rates between high- or low-dose text message groups or via feeding status.

**Table 4.** Healthy Beginnings for Hunter New England Kids click rates by feeding status.

Feeding status	High-dose group, n (%)	Low-dose group, n (%)	Difference between groups (*P* value)
Breastfeeding	15,860 (16.2)	12,084 (17.8)	.90
Mixed feeding	5361 (17)	4519 (18.7)	.89
Formula feeding	5376 (13.4)	4169 (14.4)	.92
Total	26,597 (15.7)	20,772 (17.1)	.90

#### Opt-Out Rates

Table 5 describes the opt-out rates for HB4HNEKids by phase of the program. Overall, 395 participants out of 5783 (6.8%) opted out of HB4HNEKids. Of those in the high-dose text message group, significantly more participants (241/2891, 8.3%) opted out across all phases compared to the low-dose text message group (154/2892, 5.3%). In phase 1, significantly more participants opted out of the high-dose text message group (191/2724, 7.0%) compared to the low-dose text message group (108/2812, 3.8%; *P*<.001). There were no other statistically significant differences in opt-out rates by phase or dose. There were no significant differences in opt-out rates by feeding status.

**Table 5.** Healthy Beginnings for Hunter New England Kids opt-out rates by program phase.

Phases	High-dose group, n (%)	Low-dose group, n (%)	Differences between groups (*P* value)
Phase 0 (0‐2 days)	8 (0.3)	6 (0.2)	.59
Phase 1 (2 days to 6 months)^a^	191 (7.0)	108 (3.8)	<.001
Phase 2 (6‐12 months)	10 (0.6)	16 (0.9)	.28
Phase 3 (1‐2 years)	32 (2.8)	24 (2.1)	.28
Total^a^	241 (8.3)	154 (5.3)	<.001

a Statistically significant difference in opt-out rates between high-dose and low-dose at *P*<.05.

### Acceptability by Parents and Carers

Of those that received the acceptability online survey (n=1287), 479 participants completed the survey (37% response rate). When participants were asked about the acceptability of HB4HNEKids, 84% (181/215) of high-dose participants strongly agreed or agreed compared to 85% (218/255) in the low-dose. Of high-dose participants, 85.5% (183/214) strongly agreed or agree that they were happy with how often they received text messages compared to low-dose, whereby 90.5% (228/252) agreed or strongly agreed they were happy with the frequency of messages. Among the high-dose participants, 188 of 215 (87%) and 220 of 255 low-dose participants (86%) agreed or strongly agreed that they would recommend the program to other caregivers.

### Cost

For high-dose, the average cost of sending text messages across all 3 feeding groups up to 2 years was A$12.9 per participant, whilst the average cost of sending messages for low-dose was A$9.32 per participant, making the cost difference per participant, per group A$3.64. The total cost of sending messages to families in the high-dose text message group was A$21,241.44, while the total equivalent cost for low-dose was A$15,271.48. Based on the number of participants, this is an estimated cost difference of A$5,965.96 between groups. Table 6 reports all costing of text messages per dose group.

**Table 6.** Healthy Beginnings for Hunter New England Kids text message cost per participant by dose.

Feeding groups	Cost per high-dose participant (A$)^a^	Cost per low-dose participant (A$)	Cost difference per group (A$)
Breastfeeding	13.50	9.45	4.05
Mixed feeding	13.23	9.32	3.91
Formula feeding	12.15	9.18	2.97
Average cost	12.96	9.32	3.64

a A$1 is approximately equal to US $0.68.

## Discussion

### Principal Findings

HB4HNEKids is an innovative mHealth program designed to provide theoretically developed and evidence-based age and stage appropriate information to parents in the first 2000 days. The findings of this study demonstrate the impact intervention dose has on acceptability, engagement, cost, and infant feeding outcomes in an mHealth text message–based program delivered to parents during the first 2000 days. Overall, infant feeding status and click rates did not differ by dose, with both dose groups showing high acceptability of the program. However, more participants opted out of the program from the high-dose text message group.

With feeding status not significantly changing by dose, this study demonstrated that a higher dose may not necessarily improve breastfeeding rates, which is one of the primary outcomes of the program. Click rates have been used in this study as a measure of engagement with the text messages. Of the total number of messages sent, 16% of links were clicked. With no statistically significant differences in click rates between high-dose and low-dose text message groups, it may be surmised that dose did not impact a participant’s decision to engage with the online content. The click rate for HB4HNEKids is higher than reported in other mHealth interventions of a similar nature. For example, an mHealth intervention on infant feeding and growth reported an open rate of 8% of all notifications sent, which was notably lower than HB4HNEKids [18]. However, industry rates consider the average click rate to be between 20% and 35%, which is higher than the click rates for HB4HNEKids [19]. Few mHealth programs comprehensively evaluate engagement and click rates or consider it in relation to its primary outcomes [20], making it difficult to ascertain the engagement of HB4HNEKids in comparison.

Opt-out rates were highest in HB4HNEKids in the first 6 months of the program. The first 6 months are the most intensive, with participants initially receiving 2 messages weekly for low-dose and 3‐4 messages for high. Subsequently, there was the highest level of opt-out rates in this age group, which may have been due to the intensity of the program during this phase. This pattern has also been reported in other mHealth interventions with a study on integrated health care systems reporting that those receiving more text messages were significantly more likely to opt out [21]. Acceptability of the program was considered high in both high-dose and low-dose text message groups, with the majority of participants reporting the program was acceptable and they would recommend the program to others. Interestingly, there were more participants in the low-dose text message group that considered the frequency of messages acceptable compared to those in the high-dose text message group, which needs to be considered in the context of the other study findings. When developing future content adaptations for HB4HNEKids, further consideration may need to be given if implementing higher dose messaging, in an effort to reduce opt-out rates, minimize costs, and improve acceptability of the frequency of messages.

It is important to consider this program of work in the context of its limitations. First, click rates of links within messages were used to measure engagement. Ideally, the use of open rates of text messages would be a more useful measure of engagement; however, this is a current limitation of SMS platforms. The use of web analytics may be important to explore in the future, to capture not only discrete open or click rates but to provide richer data, such as time spent on webpages, when information was accessed and whether there was repeated access. In the future, it may be useful to explore using a range of measures via a mixed-method approach to better understand the relationship between engagement and HB4HNEKids [22]. This study was conducted in the HNE region of NSW, which has an integrated public health system and experience delivering mHealth programs. These characteristics may have facilitated the implementation and engagement with the intervention. However, differences in health service organization, digital infrastructure, and population characteristics in other settings may influence the feasibility or effectiveness of similar interventions elsewhere. These contextual factors should be considered when applying the findings beyond this setting.

With infant feeding status and click rates being not significantly different based on dose and acceptability being high in both dose text message groups, the HB4HNEKids program has shown positive process outcomes, irrespective of dose. Considering opt-out rates and cost of sending messages was higher in the high-dose text message group and previous research supports the move to lowering the dose of mHealth programs long term [23], it is recommended that a lower dose of text messages may be more appropriate to use in further phases of the HB4HNEKids program as it expands across the 2‐5 year age group, and must be considered when reviewing future iterations of the program.

### Conclusions

With infant feeding status and click rates not significantly different based on dose and acceptability being high in both dose text message groups, the HB4HNEKids program has shown positive process outcomes, irrespective of dose. Considering opt-out rates and cost of sending messages was higher in the high-dose text message group, and previous research supports the move to lowering the dose of mHealth programs long term [23], it is recommended that a lower dose of text messages may be more appropriate to use in further phases of the HB4HNEKids program as it expands across the 2‐5 year age group and must be considered when reviewing future iterations of the program.

## Supplementary material

10.2196/70158 Checklist 1 CONSORT 2025 checklist.

## Abbreviations

CFHS: Child and Family Health Services
HB4HNEKids: Healthy Beginnings for Hunter New England Kids
HNE: Hunter New England
mHealth: mobile health
NSW: New South Wales
